# Application effect of apatinib in patients with failure of standard treatment for advanced malignant tumours

**DOI:** 10.1186/s40360-019-0362-2

**Published:** 2019-10-28

**Authors:** Guohui Liu, Chunbo Wang, Yunlong He, Mingyan E

**Affiliations:** Department of Radiation Oncology, The Harbin Medical University Cancer Hospital, Harbin, 150040 China

**Keywords:** Advanced malignant tumours, Apatinib, Efficacy, Safety

## Abstract

**Background:**

In recent years, targeted therapy has received widespread attention. Among these therapies, anti-angiogenic targeted drugs have become one of the hotspots of research. Apatinib is a novel oral small molecule anti-angiogenic agent that has been clinically tested in a variety of solid tumours. The aim of this study was to investigate the efficacy of apatinib in patients with advanced malignant tumours and failure of standard therapy.

**Methods:**

We collected 41 patients with advanced malignant tumours in our department; all tumours were pathologically confirmed as malignant. All patients received apatinib after failure of standard therapy: 500 mg/dose, one dose/d, orally 30 min after a meal, until progressive disease or intolerable adverse reactions occurred. When there was a second- or third-degree adverse reaction associated with apatinib during treatment, apatinib treatment could be suspended or reduced to 250 mg/dose. Clinical efficacy and progression-free survival were assessed according to RECIST1.1, and adverse reactions were observed.

**Results:**

Efficacy assessment was available for 31 patients with a median progression-free survival time of 2.66 months; the objective response rate and disease control rates were 16.1 and 64.5%, respectively. The disease control rates of the patients with lower Eastern Cooperative Oncology Group scores (1–2 points) and with fewer metastatic sites (< 3 sites) were higher than those of the patients with higher scores (3 points) and with more metastatic sites (≥3 sites), respectively (all *P* < 0.05). The most common adverse reactions were hypertension, neutropenia and hand-foot syndrome.

**Conclusion:**

For patients with advanced malignant tumours with failure of standard therapy, administration of apatinib can still result in good efficacy. The efficacy of apatinib is better in patients with a higher performance status and lower degree of tumour progression.

## Background

According to the World Health Organization (WHO), 3/5 people worldwide die from cancer, diabetes, cardiovascular disease, and chronic respiratory diseases, and cancer is one of the leading causes of death [1]. The 2018 Global Cancer Burden Status Report estimates that there were approximately 18.1 million new cancer cases and 9.6 million cancer deaths in 2018. Among all tumors, lung cancer is the most commonly diagnosed cancer and the leading cause of cancer death. The incidence of breast cancer in women, followed by the incidence of prostate cancer and colorectal cancer in men is also increasing. Cancer is the leading cause of death in China. It is also a major public health issue. Due to the large population of China, an aging population and an unhealthy lifestyle. It is expected that the incidence and mortality of cancer will increase significantly [2]..

With the deep understanding of the nature of malignant tumours and the biological behaviours and molecular biology of cancer, anticancer methods are constantly being innovated and are improving, from surgical treatment, chemotherapy, and radiation therapy to the rapid development of biological targeted therapy in recent years. Biological immunotherapy overcomes the limitations of a single treatment. In recent years, clinical scholars have proposed targeted therapy. Unlike previous surgery, radiotherapy and chemotherapy, targeted therapy has highly efficient and selective, which can greatly reduce toxic side effects and bring new hope to patients with recurrent tumors. With the rapid development of molecular biology, targeted anticancer drugs has gradually become a research hotspot for clinical scholars.

In 1971, Folkman [3] constructively suggested that tumour growth is dependent on angiogenesis. Theoretical anti-angiogenic tumour therapeutic strategies have the characteristics of a wide antitumour spectrum, resistance to drug resistance and easy access of drugs to target sites. Therefore, anti-angiogenesis-based tumour biotherapy has become a research hotspot in the past decade [4–6]. Angiogenesis plays an important role in tumor growth and metastasis [7, 8]. Anti-angiogenic drugs block the formation of new blood vessels, kill or destroy tumor blood vessels [9]. Thereby inhibiting tumor growth and metastasis [10]. The treatment of solid tumors with angiogenesis inhibitors has proven to be effective [11–14].

This study focused on the short-term efficacy and adverse effects of apatinib in patients with multiple malignancies with failure after standard treatment.

## Methods

### Clinical information

A total of 41 patients with advanced malignant tumours who were admitted to our department from July 2017 to December 2018 were pathologically confirmed to have malignant tumours. The inclusion criteria were as follows: (1) Progressive disease after treatment with a first-, second-, or third-line standard regimen of the disease (Progressive Disease, PD) or relapse; (2) age ≥ 18 years; (3) Eastern Cooperative Oncology Group (ECOG) score [15] of 0 to 3 points; (4) estimated survival time ≥ 1 month (5) Clinical stage IV; (6) measurable target lesions according to the Response Evaluation Criteria in Solid Tumours (RECIST) [16] guidelines. The exclusion criteria were as follows: (1) serious heart, liver, kidney or other organic diseases; (2) refusal to receive treatment; (3) incomplete information. Among the patients, 23 were male (5.61%), and 18 (43.9%) were female; the patients were aged 18–78 years, with a median age of 50 years. The tumour types included 10 cases of breast cancer, 7 cases of colorectal cancer, 5 cases of gastric cancer, 2 cases of non-small cell lung cancer, 3 cases of liver cancer, 2 cases of soft tissue sarcoma, 1 case of osteosarcoma, 3 cases of neuroendocrine carcinoma, and 1 case of ovarian cancer. There was 1 case of cervical cancer, 1 case of bladder cancer, 1 case of pancreatic cancer, 1 case of endometrial cancer, 1 case of primitive neuroectodermal tumour, 1 case of tongue cancer, and 1 case of metastatic adenocarcinoma. Only 2 patients (4.9%) had received adjuvant chemotherapy, 39 patients (95.1%) had received palliative chemotherapy, 11 patients (26.8%) were treated with a first-line regimen, 12 patients (29.3%) were treated with a second-line regimen and 18 were treated with a third-line or higher-line regimen (43.9%). Regarding the ECOG score, 14 patients scored 1 point (34.1%), 21 patients scored 2 points (51. 2%), and 6 patients scored 3 points (14.6%). Regarding the patients’ numbers of metastatic sites, 24 patients had < 3 metastatic sites (58.5%), and 17 patients had ≥3 metastatic sites (41.5%).

### Treatment method

All patients were treated with apatinib mesylate tablets, 500 mg/dose, one dose/d, orally 30 min after a meal, until PD or an intolerable adverse reaction occurred. When there was a second- or third-degree adverse reaction associated with apatinib during treatment, apatinib treatment could be suspended or reduced to 250 mg/dose.

### Observation index

#### Adverse reactions

All patients were followed up every week during the treatment period, and the patients’ complaints were reported. Routine blood, liver and kidney function, and urine data were reviewed. The occurrence and classification of hand-foot syndrome were observed. Adverse reactions were evaluated according to the National Cancer Institute’s General Toxicity Criteria [17] and were divided into degrees 0 to IV. The follow-up deadline was December 2018.

### Clinical efficacy

All patients underwent a full-scale imaging evaluation before treatment, and the efficacy was evaluated 3 months after administration. Clinical efficacy was evaluated according to the RECIST 1.1 [18] guidelines, including PD, Stable Disease (SD), Partial Response (PR), and Complete Remission (CR), where the Objective Response Rate (ORR) = (CR + PR) number of cases / total number of cases and the Disease Control Rate (DCR) = (CR + PR + SD) number / total number of cases.

### Progression-free survival (PFS)

Apatinib was given until tumour progression, patient loss to follow-up or time of death.

### Statistical analysis

Statistical analysis was performed using SPSS 17.0. Count data were expressed as the number of cases and percentages, and the χ2 test was used for comparisons. Survival was estimated by the Kaplan-Meier method, and the log-rank form of the χ2 test was used for comparisons. *P* < 0. 05 was considered statistically significant.

## Results

The patients’ treatment time was 0.2 ~ 15.1 months. Of the patients, 10 had not been treated for 1 month due to drug side effects or to economic or other reasons; the patients stopped taking the drug on their own, and its efficacy could not be evaluated. Therefore, efficacy could be evaluated in 31 patients. Among these patients, the rates of CR, PR, SD, and PD were 0, 5 (16.1%), 15 (48.4%), and 11 (35.5%), respectively; the ORR was 16.1%, and the DCR was 64.5%. The difference in DCR between patients with different ECOG scores and numbers of metastatic sites was statistically significant (*P* < 0.05). The DCR of patients with ECOG scores of 1 to 2 and < 3 metastatic sites was higher than that of patients with ECOG scores of ≥3 points and ≥ 3 metastatic sites, respectively (all *P* < 0.05), as shown in Table 1.

**Table 1:** Comparison of curative effects between patients with different clinical features [n (%)]

Variable	*n*	CR	PR	SD	PD	ORR	χ2	P
ECOG								
1	11	0	3(27.3)	6(54.4)	2(18.2)	3(27.3)		
2	14	0	2(14.3)	8(57.1)	4(28.6)	2(14.3)	7.731	0.021
≥ 3	6	0	0	1(16.7)	5(83.3)	0		
Sex								
Male	17	0	2(11.8)	10(58.8)	5(29.4)	2(11.8)	0.606	0.436
Female	14	0	3(21.4)	5(35.7)	6(42.9)	3(21.4)		
Chemotherapy								
Yes	2	0	1(50.0)	1(50.0)	0	1(50.0)	1.176	0.278
No	29	0	14(48.3)	14(48.3)	11(37.9)	14(48.3)		
Treatment lines								
first-line	8	0	3(37.5)	3(37.5)	4(50.0)	3(37.5)		
second-line	7	0	5(71.4)	5(71.4)	1(14.3)	5(71.4)	2.139	0.343
third-line	16	0	7(43.8)	7(43.8)	6(37.5)	7(43.8)		
metastatic sites								
< 3	17	0	11(64.7)	11(64.7)	3(17.6)	11(64.7)	5.231	0.022
≥ 3	14	0	4(28.6)	4(28.6)	8(57.1)	4(28.6)		

The median PFS time of the 31 patients was 2.66 (0. 2 to 15.1) months. There were no significant differences in the median PFS associated with ECOG score, different sex, treatment regimen, or number of metastases (both *P* > 0.05), as shown in Table 2.

**Table 2:** Comparison of median PFS time after apatinib treatment between patients with different clinical features (months)

Variable	PFS	χ2	P
ECOG			
1	2.0(0.3–15.1)		
2	2.2(0.2–3.1)	5.434	0.060
≥ 3	1.2(0.2–1.4)		
Sex			
Male	2.1(0.2–15.1)	0.753	0.326
Female	1.4(0.2–6.2)		
Treatment lines			
first-line	1.3(0.2–3.1)		
second-line	2.3(0.9–3.1)	1.932	0.246
third-line	1.5(0.2–15.1)		
metastatic sites			
< 3	2.3(0.2–15.1)	3.234	0.068
≥ 3	1.4(0.2–2.6)		

Among patients with different types of tumours, patients with neuroendocrine cancer, cervical cancer, endometrial cancer, and gastric cancer had better PFS and achieved good results, as shown in Table 3.

**Table 3:** Therapeutic effect of apatinib in different cancers

Tumor type	*n*	Efficacy evaluable number	PR(%)	SD(%)	PD(%)
Breast cancer	10	7	1(14.3)	3(42.8)	3(42.9)
NSCLC	2	2	1(50.0)	1(50.0)	0
Liver cancer	3	2	0	0	2(100.0)
Cervical cancer	1	1	0	1(100.0)	0
Osteosarcoma	1	1	0	0	1(100.0)
Soft tissue sarcoma	2	2	0	2(100.0)	0
Colorectal cancer	7	6	0	4(66.7)	2(33.3)
Ovarian cancer	1	1	0	0	1(100.0)
Neuroendocrine carcinoma	3	3	1(33.3)	2(66.7)	0
Gastric cancer	5	3	1(33.3)	2(66.7)	0
Pancreatic cancer	1	1	0	0	1(100.0)
Metastatic adenocarcinoma	1	1	0	0	1(100.0)
Bladder Cancer	1	0	0	0	0
Primitive neuroectodermal tumor	1	0	0	0	0
Tongue cancer	1	0	0	0	0
Endometrial cancer	1	1	1(100.0)	0	0

Because adverse reactions in 8 patients were unable to be followed up, 33 patients with adverse reactions were evaluated in this study. The most common adverse reactions were high blood pressure, neutropenia, and hand-foot syndrome. Among the evaluated patients, 5 patients continued to be treated for hypertension due to hypertension and 2 patients with grade III hand-foot syndrome exhibited a reduction in severity; 1 elderly patient with grade III hand-foot syndrome improved after discontinuation of the medication and did not continue taking the medication, and 1 patient died of cerebral haemorrhage due to grade IV thrombocytopenia, as shown in Table 4.

**Table 4:** Adverse reactions in patients treated with apatinib

Adverse reactions		*n*(%)	Adverse reactions		*n*(%)
Granulocyte reduction		13(39.4)	Hypertension		22(66.7)
I	II	12(36.4)	I	II	17(51.5)
III	IV	1(3.0)	III	IV	5(15.2)
Anemia		8(24.2)	Proteinuria		9(27.3)
I	II	8(24.2)	I	II	9(27.3)
III	IV	0	III	IV	0
Thrombocytopenia		1(3.0)	Hand-foot syndrome		10(30.3)
I	II	0	I	II	7(21.2)
III	IV	1(3.0)	III	IV	3(9.1)
Liver damage		3(9.1)	Weak		5(15.2)
I	II	3(9.1)	I	II	5(15.2)
III	IV	0	III	IV	0

## Discussion

Advanced malignant tumours are difficult to cure. For most types of cancer, chemotherapy is still one of the first choices for treatment. However, some patients cannot adhere to an effective chemotherapy cycle, and the efficacy of treatment and quality of life are affected due to the side effects of chemotherapy. In the choice of treatment, both efficacy and the impact of treatment on the quality of life of patients should be considered to prolong the survival time of patients with advanced cancer and improve the quality of life. With the development of precision medicine and in-depth research on cancer, targeted therapy is expected to become a new breakthrough point in the treatment of malignant tumors. Now there are some targeted drugs applied to clinical treatment, and combined with other treatment methods, which will provide a new therapeutic concept for the standard treatment of many malignancies.

Currently, targeted therapies have received extensive attention, and anti-angiogenic targeted drugs have become one of the hotspots of research. Apatinib is a small molecule tyrosine kinase inhibitor that inhibits VEGFR-2 and binds to ligands such as the vascular endothelial growth factor receptor VEGFR-2 to inhibit tumour angiogenesis [12, 14]. Compared to drugs that target VEGFR-1, apatinib, which targets VEGFR-2, has superior antiangiogenic ability. Apatinib is one of most promising antiangiogenic target drugs. Currently, apatinib has been experimentally studied in different types of cancer. The results confirmed that apatinib is an effective method for the treatment of malignant tumors. Apatinib combined with chemotherapy or targeted therapy may further improve the clinical efficacy. Moreover, related experiments show that apatinib is superior to other anti-angiogenic drugs and has good safety. Therefore, apatinib is reasonable and effective for the treatment of different advanced cancers.

In a meeting of the American Society of Clinical Oncology in 2014, some scholars reported a randomized, double-blind, placebo-controlled phase III clinical trial of apatinib as a third-line regimen for advanced gastric cancer and gastroesophageal junction tumours. As a result, the median overall survival times (4.7 and 6.5 months) and PFS times (1.8 and 2.6 months) of patients with advanced gastric cancer and gastroesophageal junction tumours, respectively, were significantly longer in the apatinib group than in the placebo group. Apatinib also showed good efficacy in phase II clinical trials of metastatic triple-negative breast cancer [19], with an ORR and DCR of 16.7 and 66.7%, respectively. In addition, apatinib is also used in other tumours, including advanced non-small cell lung cancer and advanced liver cancer. Phase II clinical studies of these tumours suggest that apatinib can significantly prolong PFS in patients and that patients exhibited different ORRs and DCRs [20]. In this study, the patients had an ORR of 16.1%, a DCR of 64.5%, and a median PFS of 2.66 months, similar to the results of the above study. Patients with multiple advanced malignancies and treatment failure after the standard regimen who were treated with apatinib still exhibited improved results. In addition, for patients with ECOG scores of 1 to 2 and < 3 metastatic sites, these rates were higher than those for patients with ECOG scores of ≥3 and ≥ 3 metastases (both *P* < 0.05), indicating that these patients’ physical status was improved. The lower the degree of tumour progression, the better was the therapeutic effect of apatinib.

Studies have shown that apatinib can inhibit efflux pumping mediated by P-glycoprotein (ATP-binding cassette subfamily B member 1 transporter gene, ABCB1), multidrug resistance associated protein 1 (MRP1), ATP-binding cassette subfamily C member 1 Transporter gene (ABCC1), breast cancer resistance protein (BCRP), and ATP-binding cassette sub-family G member 2 (ABCG2) to reverse multidrug resistance in solid tumour cells [21]. In addition, apatinib can also downregulate the phosphorylation of extracellular signal-regulated kinase 1/2 and the phosphorylation of protein kinase B, induce the apoptosis and inhibit the proliferation of multidrug-resistant HL-60 leukaemia cells, and increase the killing effect of paired doxorubicin treatment on leukaemia cells with high expression of ABCB-1 [22, 23]. In this study, the ORR, DCR, and PFS of patients treated with second- and third-line regimens were slightly higher than those treated with the first-line regimen, suggesting that patients with drug resistance to second- and third-line chemotherapy may still be able to obtain a good treatment effect with apatinib. However, there was no significant difference in the DCR or PFS time between patients treated with different regimens (*P* > 0.05), which may be related to the small sample size. It is worth noting that anti-angiogenic drugs also have the problem of drug resistance, which may render them ineffective after a period of treatment. In the case of tumour recurrence, this effect may be due to tumour-independent VEGFR signalling pathways, which are compensated by other signalling pathways. There are also many clinical trials that have attempted to combine apatinib with chemotherapy to control drug resistance.

Among different types of tumours, neuroendocrine cancer has a relatively high degree of malignancy and is prone to recurrence and metastasis. Currently, the main treatment is surgery. Advanced patients are relatively resistant to chemotherapy, and octreotide and everolimus are considered. Sunitinib and others are given as first-line drugs. Of the 3 patients with neuroendocrine carcinoma in this study, 1 patient progressed with the third-line regimen and achieved partial remission after treatment with apatinib. The PFS time was 11.5 months. The other 2 patients maintained stable disease with a PFS time of > 9 months. These 3 patients were still taking the drug; 1 patient with endometrial cancer was partially relieved, and the PFS time was 4 .6 months; 1 patient with cervical cancer also achieved stable disease and maintained PFS for 6.2 months; this patient is currently on maintenance medication. This result suggests that apatinib can achieve better results in the abovementioned tumours than in other tumour types. Currently, there is no effective targeted therapy for triple-negative breast cancer, and chemotherapy is still the main treatment. This study included 10 patients with advanced breast cancer, 7 of whom had advanced triple-negative breast cancer. After application of apatinib, 1 patient was partially relieved, 3 patients achieved stable disease, and the median PFS time was 1.1 months. Thus, apatinib may be an effective treatment for patients with triple-negative breast cancer who have limited treatment options and poor prognosis. Due to the wide coverage of the disease types in this study, the sample size of each disease type was small, and the value of the analysis results is limited. In subsequent studies, the sample size could be considered for further expansion, or a single disease type could be further studied.

In this study, the most common adverse events were hypertension, neutropenia, and hand-foot syndrome, similar to those reported in other literature [14]. However, most patients can continue taking the drug after reduction or withdrawal, and the tolerance is good. Among our patients, 1 patient, who was treated with capecitabine while taking apatinib, died of cerebral haemorrhage due to stage IV thrombocytopenia. There is no definitive basis to prove that apatinib combined with chemotherapy is superior to single-agent therapy, and a rigorous clinical trial is needed to explore the efficacy and safety of this regimen and to find a suitable group for chemotherapy.

## Conclusion

In summary, treatment of patients with advanced malignant tumours and failure of standard treatment can still achieve improved results. The better the patient’s physical state and the lower the degree of tumour progression, the better is the therapeutic effect of apatinib.

## Data Availability

All data and materials are fully available without restriction.

## Abbreviations

ABCB1: ATP-binding cassette subfamily B member 1 transporter gene
BCRP: Breast Cancer Resistance Protein
CR: Complete Remission
DCR: Disease Control Rate
ECOG: Eastern Cooperative Oncology Group
GC: Gastric Cancer
MRP1: Multidrug Resistance Associated Protein 1
ORR: Objective Response Rate
PD: Progressive Disease
PFS: Progression-free survival
PR: Partial Response
RECIST: Response Evaluation Criteria in Solid Tumours
SD: Stable Disease
VEGF: Vascular Endothelial Growth Factor
VEGFR: Vascular Endothelial Growth Factor Receptor

## References

[1] Fidler MM, Soerjomataram I, Bray F (2016). A global view on cancer incidence and national levels of the human development index. Int J Cancer.

[2] Chen W, Xia C, Zheng R, Zhou M, Lin C, Zeng H, Zhang S, Wang L, Yang Z, Sun K, Li H (2019). Disparities by province, age, and sex in site-specific cancer burden attributable to 23 potentially modifiable risk factors in China: a comparative risk assessment. Lancet Glob Health.

[3] Folkman J. Tumor angiogenesis: therapeutic implications. N Engl J Med. 1971, 285;(21):1182–6. 10.1056/NEJM197111182852108 4938153

[4] Tian S, Quan H, Xie C (2011). YN968D1 is a novel and selective inhibitor of vascular endothelial growth factor receptor-2 tyrosine kinase with potent activity in vitro and in vivo. Cancer Sci.

[5] Yewale C, Baradia D, Vhora I, Patil S, Misra A (2013). Epidermal growth factor receptor targeting in cancer:a review of trends and strategies. Biomaterials..

[6] Ferrara N, Kerbel RS (2005). Angiogenesis as a therapeutic target. Nature.

[7] Ferrara N, Adamis AP (2016). Ten years of anti-vascular endothelial growth factor therapy. Nat Rev Drug Discov.

[8] Simons M, Gordon E, Claesson-Welsh L (2016). Mechanisms and regulation of endothelial VEGF receptor signalling. Nat Rev Mol Cell Biol.

[9] Koch S, Tugues S, Li X (2011). Signal transduction by vascular endothelial growth factor receptors. Biochem J.

[10] Ferrara N, Gerber HP, Lecouter J (2003). The biology of VEGF and its receptors. Nat Med.

[11] Li J, Qin S, Xu J (2016). Randomized, double-blind, placebo-controlled phase III trial of apatinib in patients with chemotherapy-refractory advanced or metastatic adenocarcinoma of the stomach or gastroesophageal junction. J Clin Oncol.

[12] Huang L, Wei Y, Shen S (2017). Therapeutic effect of apatinib on overall survival is mediated by prolonged progression-free survival in advanced gastric cancer patients. Oncotarget..

[13] Scott LJ (2018). Apatinib: a review in advanced gastric cancer and other advanced cancers. Drugs.

[14] Chen LT, Oh DY, Ryu MH (2017). Anti-angiogenic therapy in patients with advanced gastric and gastroesophageal junction cancer: a systematic review. Cancer Res Treat.

[15] Park CM, Koh Y, Jeon K (2014). Impact of eastern cooperative oncology group performance status on hospital mortality in critically ill patients. J Crit Care.

[16] Schwartz LH, Seymour L, Litière S (2016). RECIST 1.1—standardisation and disease-specific adaptations: perspectives from the RECIST working group. Eur J Cancer.

[17] Lerner SP, Jarow J, Maher VE, Tang S, Ibrahim A, Kim G (2015). Development of systemic and topical drugs to treat non-muscle invasive bladder Cancer. Bl Cancer.

[18] Nishino M, Jackman DM, Hatabu H (2010). New Response Evaluation Criteria in Solid Tumors( RECIST) guidelines for advanced non-small cell lung cancer: comparison with original RECIST and impact on assessment of tumor response to targeted therapy. AJR Am J Roentgenol.

[19] Hu X, Zhang J, Xu B (2014). Multicenter phase II study of apatinib, a novel VEGFR inhibitor in heavily pretreated patients with metastatic triple-negative breast cancer. Int J Cancer.

[20] Qin SK (2014). Apatinib in Chinese patients with advanced hepatocellular carcinoma: a phase II randomized, open-label trial. J Clin Oncol.

[21] Merriman TR (2015). An update on the genetic architecture of hyperuricemia and gout. Arthritis Res Ther.

[22] Köttgen A, Albrecht E, Teumer A (2013). Genome-wide association analyses identify 18 new loci associated with serum urate concentrations. Nat Genet.

[23] Tong XZ, Wang F, Liang S (2012). Apatinib( YN968D1) enhances the efficacy of conventional chemotherapeutical drugs in side population cells and ABCB1-overexpressing leukemia cells. Biochem Pharmacol.

